# Neutrophil count is associated with survival in localized prostate cancer

**DOI:** 10.1186/s12885-015-1599-9

**Published:** 2015-08-21

**Authors:** Houda Bahig, Daniel Taussky, Guila Delouya, Amal Nadiri, Ariane Gagnon-Jacques, Paule Bodson-Clermont, Denis Soulieres

**Affiliations:** 1 Department of Radiation Oncology, Centre Hospitalier de l’Université de Montréal (CHUM), Hôpital Notre-Dame, Montreal, Canada; 2 Medical Oncology, Centre Hospitalier de l’Université de Montréal (CHUM), Hôpital Notre-Dame, Montreal, Canada; 3 CRCHUM-Centre de recherche du Centre Hospitalier de l’Université de Montréal, Montreal, Canada

## Abstract

**Background:**

Increasing evidence suggests a close relationship between systemic inflammation and cancer development and progression. The neutrophil to lymphocyte ratio (NLR) has been shown to be an independent prognostic indicator in various advanced and localized cancers. We investigated the influence of markers of systemic inflammation such as leucocyte counts and metabolic co-morbidities on overall survival (OS) after radiotherapy for localized prostate cancer.

**Methods:**

We conducted a retrospective study of patients with localized prostate cancer treated with definitive external beam radiotherapy or brachytherapy. Univariate and multivariate cox proportional hazards models were used to investigate the influence of the following factors on OS: age, neutrophil and lymphocyte counts, neutrophil-to-lymphocyte ratio (NLR), Cancer of the Prostate Risk Assessment (CAPRA) score as well as comorbidities associated with inflammation such as cardiac history, diabetes and use of a statin. A stepwise selection of variable based on the Akaike information criterion (AIC) was used for multivariate analysis.

**Results:**

In total, 1772 pts were included; blood count data was available for 950 pts. Median age was 68 years (44–87). Actuarial 5 years OS and biochemical recurrence-free survival (BRFS) for the 1772 patients were 93 % and 95 %, respectively, with a median follow-up of 44 months (1–156). On univariate analysis, neutrophil count (p = 0.04), cardiac history (p = 0.008), age (p = 0.001) and CAPRA (p = 0.0002) were associated with OS. Lymphocytes, NLR and comorbidities other than cardiac history were not associated with mortality. On multivariate analysis, neutrophil count (HR = 1.18, 95 % CI: 1.017-1.37, p = 0.028), age (HR = 1.06, 95 % CI: 1.01-1.1, p = 0.008) and CAPRA (HR = 1.16, 95 % CI: 1.03-1.31, p = 0.015) were independent predictors of OS.

**Conclusion:**

Neutrophil count, as a possible marker of systemic inflammation, appear to be an independent prognostic factor for overall mortality in localized prostate cancer. A validation cohort is needed to corroborate these results.

## Background

Inflammation is a fundamental innate immune response associated with disruption of homeostasis. Increasing evidence suggests a close relationship between systemic inflammation and cancer development and progression [1–4]. Inflammatory leukocytes such as neutrophils, monocytes, macrophages, and eosinophils provide the soluble factors that are thought to mediate the development of inflammation-associated cancer. In recent years, several readily measurable peripheral blood markers such as the neutrophil to lymphocyte ratio (NLR) [5–10], the Glasgow Prognostic score (consisting in a combination of C-reactive protein and albumin level) [11–13], the platelet to lymphocyte ratio [14, 15] or the Prognostic index (consisting in white blood cell count and C-reactive protein) [16], were associated with cancer outcomes in large cohort studies. More specifically, the NLR has been recognized as an independent prognostic indicator in various advanced and localized cancer including gastro-intestinal cancers [6, 9, 17–21], urological cancers [5, 10, 22, 23], lung cancers [8, 24], head and neck cancers [7, 25], ovarian cancers [8], and glioblastomas [7]. In the case of prostate cancer, studies have reported that NLR was associated with treatment response and survival in patients with metastatic castrate resistant prostate cancer receiving systemic therapy [22, 26]. However, the prognostic value of markers of systemic inflammation was never previously evaluated in the context of localized prostate cancer.

The following study was designed to explore the influence of readily available markers of systemic inflammation such as leucocyte count and metabolic co-morbidities associated with systemic inflammation on biochemical recurrence-free survival (BRFS) and overall survival (OS) after curative radiotherapy for localized prostate cancer in a large cohort of patients.

## Methods

### Patients’ characteristics

Institutional review board (IRB) approval from the Centre Hospitalier de l’Université de Montréal (CHUM) was obtained for this study (Ref 14.144). This being a retrospective study, informed consent was waived by the IRB.

A review of our institutional database of patients with localized prostate cancer treated with definitive external beam radiotherapy or brachytherapy from September 2001 to June 2014 was conducted. Inclusion criteria were: (1) Eastern Cooperative Oncology Group performance status < 2, (2) histology proven prostate cancer; (2) localized prostate cancer stage T1-3bN0M0 as per the American Joint Committee on Cancer 7th edition; (3) brachytherapy or external beam radiotherapy(EBRT) or a combination of both with curative intent. Prostate cancer risk group was defined as per the National Comprehensive Cancer Network (NCCN) Guidelines classification [27]. Patients with evidence of radiological or histological lymph node involvement or distant metastasis were excluded. Patients receiving androgen deprivation therapy were included. Initial work-up at diagnosis included: medical history including presence of co-morbidities, physical examination including digital rectal examination, complete blood count and biochemistry, serum prostate specific antigen (PSA), transrectal ultrasound and biopsy. For patients with stage T3-4 disease, PSA ≥10 ng/mL or a Gleason score (GS) ≥7, staging bone scan and pelvic computed tomography (CT) were done at the treating physician’s discretion to exclude distant metastasis.

### Treatment characteristics

All patients were treated with curative radiotherapy, with or without androgen deprivation therapy. Low dose rate (LDR) brachytherapy alone with Iodine^125^ permanent seeds to a dose of 144 grays (Gy) was considered for low-risk prostate cancer or intermediate-risk prostate cancer with favorable features. Combined brachytherapy and EBRT (LDR dose of 110 Gy and EBRT dose of 44 Gy in 22 fractions versus HDR dose of 15 Gy in 1 fraction and EBRT dose of 37.5 Gy in 15 fractions) was considered for intermediate or high-risk prostate cancer. When EBRT was given as exclusive therapy, a dose of 70 to 80 Gy was given with or without hormonal therapy.

### Follow-up and statistics

Standard follow-up for all patients typically included: physical examination and serum PSA every 3 months for the first 2 years, every 6 months for the following 3 years, and annually thereafter. Biochemical failure was defined as per the Phoenix consensus (nadir + 2 ng/mL) [28]. Follow-up duration was defined as the time from the date of treatment completion to the date of last follow-up or death. Kaplan-Meier method was used for estimation of OS and BRFS. Univariate and multivariate Cox proportional hazards models analysis were performed to determine predictors of OS and BRFS. Potential baseline predictive factors explored included: age, UCSF Cancer of the prostate risk assessment score (CAPRA) (combining age, PSA, Gleason score, clinical stage and percent positive biopsy cores), body mass index (BMI), metabolic co-morbidities associated with systemic inflammation (cardiovascular disease, diabetes, hypertension, use of statins), as well as leucocyte counts (neutrophils, lymphocytes and neutrophil-to-lymphocyte ratio). Multivariate analysis was carried using stepwise regression method. A stepwise selection of variable based on the Akaike information criterion (AIC) was used for multivariate analysis to determine the best fit model [29]. Statistical analyses were conducted using SPSS 17 (SPSS, Chicago, IL), SAS 9.4 (SAS Institute, Cary, NC) and R version 2.15.2. All reported p values were considered to be statistically significant at <0.05 from two-sided tests.

## Results

### Patients’ characteristics

In total, 1772 patients were included in the analysis; blood count data was available for 950 patients. Median age was 68 years (range: 44–87). The majority of patients (64 %) presented with stage T1 disease. Low, intermediate and high risk prostate cancer constituted respectively 41 %, 49 % and 10 % of the cohort. In total, 42 % of patients presented a CAPRA score of 0–2, 46 % of 3–5 and 12 % of 6–10. Patients’ characteristics including metabolic co-morbidities and leucocyte counts as well as treatment characteristics are summarized in Table 1. Comorbidities included diabetes in 15.1 % of patients, cardiovascular disease in 34.6 % and hypertension in 45.7 %. In addition, 42.6 % of patients used a statin for hypercholesterolemia.

**Table 1:** Patients and treatments characteristics

Age (y)		
Median (range)	67	44-87
PSA at diagnosis (ng/mL)		
Median (range)	8.1	0.2-132
AJCC tumor classification (N, %)		
T1	1126	64 %
T2	627	32 %
T3	80	4 %
Gleason score (N; %)		
≤6	930	53 %
7	752	42 %
≥8	90	5 %
NCCN risk groups (N; %)		
Low	748	42 %
Intermediate	855	48 %
High	169	10 %
PPC (N, %)		
<34 %	883	50 %
≥34 %	883	50 %
CAPRA score>		
0-2	759	43 %
3-5	811	46 %
6-10	194	11 %
BMI		
Median (range)	28.2	13.9–52.5
Diabetes (N; %)>		
Yes	272	15 %
No	1500	85 %
Statin use (N; %)>		
Yes	762	43 %
No	1010	57 %
CVD (N; %)		
Yes	629	35 %
No	1143	65 %
HTN (N; %)		
Yes	815	46 %
No	957	54 %
Lymphocytes (10^3^ cells/μL)		
Median (range)	1.7	0.4–16
Neutrophils (10^3^ cells/μL)		
Median (range)	4.5	0.9–12
NLR		
Median (range)	3.0	0.1–18.4
*Treatment characteristics*		
Radiotherapy		
HDR brachytherapy	52	3 %
LDR brachytherapy	901	51 %
EBRT	815	46 %
Androgen deprivation>		
Non	1551	87 %
Yes	221	13 %

PSA = prostate-specific antigen; AJCC = American Joint Committee on Cancer; NCCN = National Comprehensive Cancer Network; PPC = percentage of positive cores at prostate biopsies; CAPRA = Cancer of the Prostate Risk Assessment score, BMI = body mass index; CVD = cardiovascular disease; HTN = hypertension. >HDR = High dose rate; LDR = Low dose rate. Lymphocytes, neutrophils and NLR were available for 950 patients; BMI was available for 544 patients; data was available for entire cohort (1772 patients) for all the other factors.

### Biochemical and overall survival outcomes and predictive factors

**Fig. 1:**
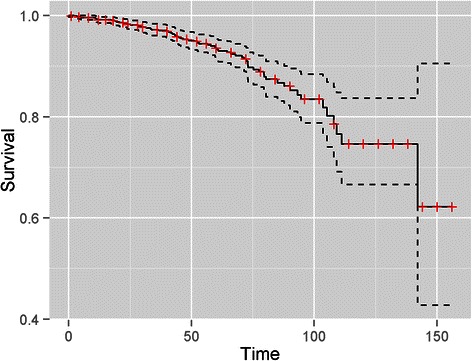
Kaplan Meier OS curve with lower and upper limits of the 95 % confidence intervals showing a 5-year OS of 93 % (95 % CI = 91–95 %) in patients with localized prostate cancer treated with radical radiotherapy or brachytherapy

**Fig. 2:**
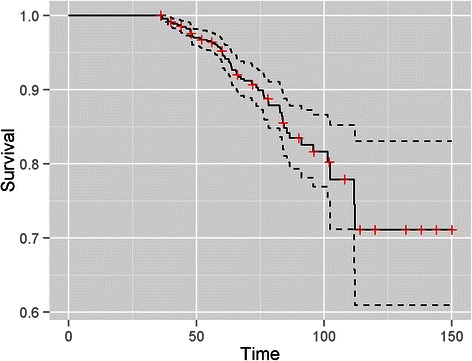
Kaplan Meier BRFS curve with lower and upper limits of the 95 % confidence intervals showing a 5-year BFRS of 95 % (95 % CI = 93–96 %) in patients with localized prostate cancer treated with radical radiotherapy

Actuarial 5 years OS and BRFS for the entire cohort (1772 patients) was respectively 93 % and 95 %, with a median follow-up of 44 months (range: 1–156) (Figs. 1 and 2). On univariate analysis, neutrophil count (p = 0.04), cardiac history (p = 0.009), age (p = 0.001) and CAPRA (p = 0.0002) were associated with OS (Table 3). Lymphocytes, NLR and comorbidities other than cardiac history were not associated with mortality. On multivariate analysis, neutrophil count (HR = 1.18, 95 % CI: 1.017-1.37, p = 0.028), age (HR = 1.06, 95 % CI: 1.01-1.1, p = 0.008) and CAPRA (HR = 1.16, 95 % CI: 1.03-1.31, p = 0.015) were independent predictors of OS (Table 2).

**Table 2:** Univariate and multivariate analysis of factors associated with OS

	Univariate		Multivariate	
	HR (95 % CI)	p-value	HR (95 % CI)	p-value
Age (n = 1772)	1.06 (1.03 1.10)	0.001	1.05 (1.01–1.10)	0.008
CAPRA (n = 1772)	1.21 (1.09–1.34)	0.0002	1.16 (1.03–1.30)	0.015
BMI (n = 544)	1.00 (0.93–1.06)	0.9		
CVD (n = 1772)	1.79 (1.16–2.77)	0.009		
HTN (n = 1772)	1.14 (0.74–1.76)	0.5		
Diabetes (n = 1772)	1.59 (0.94–2.68)	0.08		
Statin use (n = 1772)	0.82 (0.52–1.28)	0.4	0.70 (0.42–1.14)	0.15
Neutrophils (n = 950)	1.16 (1.01–1.34)	0.04	1.18 (1.017–1.37)	0.03
Lymphocytes (n = 950)	1.01 (0.72–1.43)	0.9		
NLR (n = 950)	1.10 (0.95–1.27)	0.2		
WBC (n = 950)	1.13 (0.99–1.28)	0.06		

n = number of patients with available data

WBC = white blood count (=Neutrophils + Lymphocytes)

On univariate analysis, only CAPRA was associated with BRFS (p = 0.0001). On multivariate analysis, both age (HR = 0.94, 95 % CI 0.91-0.98, p = 0.009) and CAPRA (HR = 1.26, 95 % CI 1.12-1.43, p = 0.0002) were associated with BRFS (Table 3).

**Table 3:** Univariate and multivariate analysis of factors associated with BRFS

	Univariate		Multivariate	
	HR (95 % CI)	p-value	HR (95 % CI)	p-value
Age (n = 1041)	0.98 (0.94–1.01)	0.2	0.94 (0.91–0.98)	0.009
CAPRA (n = 1041)	1.23 (1.11–1.37)	0.0001	1.26 (1.12–1.43)	0.0002
BMI (n = 372)	1.02 (0.97–1.08)	0.5		
CVD (n = 1044)	0.73 (0.46–1.16)	0.2		
Diabetes (n = 1044)	0.86 (0.44–1.67)	0.7		
Statin use (n = 1044)	0.79 (0.50–1.24)	0.3	0.67 (0.39–1.17)	0.2
Neutrophils (n = 608)	1.04 (0.87–1.23)	0.7		
Lymphocytes (n = 609)	0.98 (0.64–1.48)	0.9		
NLR (n = 608)	1.02 (0.85-1.22)	0.9		

## Discussion

To our knowledge, this is the first study in the literature reporting an association between neutrophil count and prognosis in localized prostate cancer. In our large study cohort, neutrophil counts were found to be associated with worse OS outcomes. However, such an association was not found for BRFS nor was it found for other markers such as NLR. In addition, in concordance with previous literature [30, 31], chronological age as well as CAPRA- score as indicator of cancer aggressiveness, were expectedly associated with OS.

The association of neutrophils with survival found in this study is in agreement with the now established association between systemic inflammation and cancer outcomes in many different cancers [1, 32–34], and the increasing evidence supporting the prognostic importance of neutrophil count as a marker of inflammation [23, 35, 36]. The prognostic significance of neutrophil counts was recently suggested in metastatic prostate cancer. In fact, in a first study by Keizman [26] where the association between pretreatment NLR and cancer outcome in 156 patients with metastatic castration-resistant prostate cancer treated with ketoconazole was assessed, pretreatment NLR >3 was found to be associated with worse progression-free survival (PFS) but was not associated with OS. In a second retrospective study including 33 patients with metastatic castrate resistant prostate cancer treated with docetaxel and prednisone [37], an increased NLR was found to be associated with a decreased PSA response.

### Inflammation in cancer and neutrophil count

How neutrophils affect cancer prognosis remain unclear. Systemic inflammation is believed to be associated with cancer progression through several mechanisms, including: cancer cell development, proliferation and survival, angiogenesis, metastatic potential increase and diminished response to therapy [2]. In metastatic prostate cancer, molecular signaling triggered by inflammatory mediators was hypothesized to promote cancer cell progression [38]. Neutrophils are hypothesized to contribute to tumor progression and aggressive biology through the production of cytokines (such as such as tumor necrosis factor, interleukin (IL)-1, and IL-6), chemokines and granule proteins, which are thought to promote tumor proliferation and angiogenesis, and increase its metastatic potential [18, 39, 40]. On the other hand, the adverse prognosis associated with high NLR could also partly be explained by a lower lymphocyte count which can reflect a decreased anti-tumor immune response, an increased neutrophil response to the tumor and which consequently favors angiogenesis and tumor progression or both [41].

This being a retrospective study, we were unable to include other known factors of inflammation such as C-reactive protein or albumin levels in this study, because they are not routinely included in laboratory investigations of localized prostate cancer. Neutrophil count and NLR have the advantage of being inexpensive and readily available markers of systemic inflammation. However, although numerous studies support their prognostic role, cut-off values vary significantly among studies, complicating its current use in the clinical setting.

### Neutrophil count as prognostic indicator in cancer

Several studies have reported an association between increased peripheral neutrophil count and prognosis in numerous cancers, predominantly locally advanced or metastatic. In a study by Teramukai [36], increased neutrophil count was associated with both decreased OS and PFS in patients with stage IIIB and IV non-small cell lung cancer. Similarly, EORTC 18951 trial showed that both pre-treatment neutrophil and leukocyte counts were independent prognostic factor for OS in patients with metastatic melanoma undergoing immunotherapy [35]. In addition, a high baseline NLR was found to be a predictor of poor prognosis in many cancers treated with systematic therapy including: malignant mesothelioma [42], colorectal cancer metastatic to liver [17], pancreatic cancer [6], gastric cancer [20] or advanced esophageal cancer [21].

One could assume that increased peripheral neutrophils would be associated with worse outcomes in systemic disease only, but numerous studies have reported similar results in localized cancers. In fact, in patients undergoing radical surgery for limited stage cancers, high pre-operative NLR was associated with both worse OS and DSS in colorectal cancer [43], hepatocellular carcinoma [9], bladder cancer [10] renal carcinoma [5], gastric cancer [44], and early stage non-small cell lung cancer [24]. In addition, increased baseline NLR was associated with poorer OS in patients undergoing treatment for glioblastoma multiforme [7] and nasopharygeal cancer [25]. In our study, neutrophils were predictive of OS but no such association was found with BRFS. In addition, NLR was associated with neither OS nor BRFS. It is therefore difficult to determine whether systemic inflammation was the cause for cancer progression or simply a marker for systemic inflammation, which could be a cause for an increase in mortality. The number of deaths was too small to stratify according to cause of death in order to determine whether patients with high neutrophils were more likely to die from cardiac problems or prostate cancer. This will be the subject of a later analysis after longer follow-up.

### Other markers of systemic inflammation

Many studies have looked at other markers of systemic inflammation. In fact, C-reactive protein alone [32], the Glasgow prognostic score constituting a combination of C-reactive protein and albumin levels [12], the platelet to lymphocyte ratio [14, 15] or the Prognostic index constituted of a combination of C-reactive protein and white cell count [11], were all shown to be associated with survival in cancer patients. Independent of tumor stage, the Glasgow prognostic score has comprehensively been validated to be prognostic in lung, gastro-intestinal and renal cancers [3]. In a large cohort of 27 031 cancer patients, Proctor at al. [13] reported that both the Glasgow score and the Prognostic index had similar prognostic value in cancer, independent of cancer type. Interestingly, their results suggested that rather than the NLR, leucocytes may be best useful addition to the Glasgow score.

Limitations of our study include its retrospective nature with associated selection bias, possible confounding factors and missing data. As leukocyte data was not available for all patients, our study may have been underpowered to detect a statistically significant difference in NLR or lymphocyte counts. Additional known inflammatory markers such as C-reactive protein and albumin level were not routinely available and therefore could not be included in the analysis. In addition, the small number of events and lack of validation cohort precluded the decision to determine a clinically useful neutrophil and age cut-off based on this cohort.

However, we believe that results from this large cohort of patients should be considered at time of treatment decision, especially in older patients. Readily available inflammatory marker such as neutrophil count o better predict survival outcomes and therefore guide optimal treatment decision-making. In fact, a significant proportion of localized prostate cancer is diagnosed in an older population of patients aged ≥ 70 years. For these patients, treatment aggressiveness can vary widely, from watchful waiting to radical local treatment associated with systemic androgen deprivation therapy, depending on their predicted survival outcomes. In addition, a better understanding of the mechanism by which these inflammatory markers contribute to poorer survival may open the door to new targets of systemic therapies.

## Conclusion

Overall mortality in localized prostate cancer depends not only on age and cancer aggressiveness, but also on neutrophil count as a marker of systemic inflammation. Neutrophil counts may prove useful in treatment decision algorithm of these patients. A validation cohort is needed to corroborate these results.

## Abbreviations

NLR: Neutrophil to lymphocyte ratio
OS: Overall survival
CAPRA: Cancer of the prostate risk assessment
AIC: Akaike information criterion
FRS: Biochemical recurrence-free survival
HR: Hazard ratio
CI: Confidence interval
NCCN: National comprehensive cancer network
PSA: Prostate specific antigen
GS: Gleason score
CT: Computed tomography
LDR: Low dose rate
Gy: Grays
EBRT: External beam radiotherapy
BMI: Body mass index
PFS: Progression-free survival
IL: Interleukin
DSS: Disease-specific survival

## References

[1] Coussens LM, Werb Z (2002). Inflammation and cancer. Nature.

[2] Mantovani A, Allavena P, Sica A, Balkwill F (2008). Cancer-related inflammation. Nature.

[3] McMillan DC (2009). Systemic inflammation, nutritional status and survival in patients with cancer. Curr Opin Clin Nutr Metab Care.

[4] Balkwill F, Charles KA, Mantovani A (2005). Smoldering and polarized inflammation in the initiation and promotion of malignant disease. Cancer Cell.

[5] Ohno Y, Nakashima J, Ohori M, Hatano T, Tachibana M (2010). Pretreatment neutrophil-to-lymphocyte ratio as an independent predictor of recurrence in patients with nonmetastatic renal cell carcinoma. J Urol.

[6] An X, Ding PR, Li YH, Wang FH, Shi YX, Wang ZQ, He YJ, Xu RH, Jiang WQ (2010). Elevated neutrophil to lymphocyte ratio predicts survival in advanced pancreatic cancer. Biomarkers.

[7] Bambury RM, Teo MY, Power DG, Yusuf A, Murray S, Battley JE, Drake C, O'Dea P, Bermingham N, Keohane C (2013). The association of pre-treatment neutrophil to lymphocyte ratio with overall survival in patients with glioblastoma multiforme. J Neurooncolsss.

[8] Cho H, Hur HW, Kim SW, Kim SH, Kim JH, Kim YT, Lee K (2009). Pre-treatment neutrophil to lymphocyte ratio is elevated in epithelial ovarian cancer and predicts survival after treatment. Cancer Immunol Immunother.

[9] Gomez D, Farid S, Malik HZ, Young AL, Toogood GJ, Lodge JP, Prasad KR (2008). Preoperative neutrophil-to-lymphocyte ratio as a prognostic predictor after curative resection for hepatocellular carcinoma. World J Surg.

[10] Gondo T, Nakashima J, Ohno Y, Choichiro O, Horiguchi Y, Namiki K, Yoshioka K, Ohori M, Hatano T, Tachibana M (2012). Prognostic value of neutrophil-to-lymphocyte ratio and establishment of novel preoperative risk stratification model in bladder cancer patients treated with radical cystectomy. Urology.

[11] McMillan DC (2013). The systemic inflammation-based Glasgow Prognostic Score: a decade of experience in patients with cancer. Cancer Treat Rev.

[12] Proctor MJ, Morrison DS, Talwar D, Balmer SM, O'Reilly DS, Foulis AK, Horgan PG, McMillan DC (2011). An inflammation-based prognostic score (mGPS) predicts cancer survival independent of tumour site: a Glasgow Inflammation Outcome Study. Br J Cancer.

[13] Proctor MJ, Morrison DS, Talwar D, Balmer SM, Fletcher CD, O'Reilly DS, Foulis AK, Horgan PG, McMillan DC (2011). A comparison of inflammation-based prognostic scores in patients with cancer. A Glasgow Inflammation Outcome Study. Eur J Cancer.

[14] Smith RA, Bosonnet L, Raraty M, Sutton R, Neoptolemos JP, Campbell F, Ghaneh P (2009). Preoperative platelet-lymphocyte ratio is an independent significant prognostic marker in resected pancreatic ductal adenocarcinoma. Am J Surg.

[15] Smith RA, Ghaneh P, Sutton R, Raraty M, Campbell F, Neoptolemos JP (2008). Prognosis of resected ampullary adenocarcinoma by preoperative serum CA19-9 levels and platelet-lymphocyte ratio. J Gastrointest Surg.

[16] Kasymjanova G, MacDonald N, Agulnik JS, Cohen V, Pepe C, Kreisman H, Sharma R, Small D (2010). The predictive value of pre-treatment inflammatory markers in advanced non-small-cell lung cancer. Curr Oncol.

[17] Kishi Y, Kopetz S, Chun YS, Palavecino M, Abdalla EK, Vauthey JN (2009). Blood neutrophil-to-lymphocyte ratio predicts survival in patients with colorectal liver metastases treated with systemic chemotherapy. Ann Surg Oncolss.

[18] Kuang DM, Zhao Q, Wu Y, Peng C, Wang J, Xu Z, Yin XY, Zheng L (2011). Peritumoral neutrophils link inflammatory response to disease progression by fostering angiogenesis in hepatocellular carcinoma. J Hepatol.

[19] Liu H, Liu G, Bao Q, Sun W, Bao H, Bi L, Wen W, Liu Y, Wang Z, Yin X (2010). The baseline ratio of neutrophils to lymphocytes is associated with patient prognosis in rectal carcinoma. J Gastrointest Cancer.

[20] Yamanaka T, Matsumoto S, Teramukai S, Ishiwata R, Nagai Y, Fukushima M (2007). The baseline ratio of neutrophils to lymphocytes is associated with patient prognosis in advanced gastric cancer. Oncology.

[21] Sato H, Tsubosa Y, Kawano T (2012). Correlation between the pretherapeutic neutrophil to lymphocyte ratio and the pathologic response to neoadjuvant chemotherapy in patients with advanced esophageal cancer. World J Surg.

[22] Keizman D, Ish-Shalom M, Huang P, Eisenberger MA, Pili R, Hammers H, Carducci MA (2012). The association of pre-treatment neutrophil to lymphocyte ratio with response rate, progression free survival and overall survival of patients treated with sunitinib for metastatic renal cell carcinoma. Eur J Cancer.

[23] Donskov F, von der Maase H (2006). Impact of immune parameters on long-term survival in metastatic renal cell carcinoma. J Clin Oncol.

[24] Sarraf KM, Belcher E, Raevsky E, Nicholson AG, Goldstraw P, Lim E (2009). Neutrophil/lymphocyte ratio and its association with survival after complete resection in non-small cell lung cancer. J Thorac Cardiovasc Surgss.

[25] An X, Ding PR, Wang FH, Jiang WQ, Li YH (2011). Elevated neutrophil to lymphocyte ratio predicts poor prognosis in nasopharyngeal carcinoma. Tumour Biol.

[26] Keizman D, Gottfried M, Ish-Shalom M, Maimon N, Peer A, Neumann A, Rosenbaum E, Kovel S, Pili R, Sinibaldi V (2012). Pretreatment neutrophil-to-lymphocyte ratio in metastatic castration-resistant prostate cancer patients treated with ketoconazole: association with outcome and predictive nomogram. Oncologist.

[27] Mohler J, Bahnson RR, Boston B, Busby JE, D'Amico A, Eastham JA, Enke CA, George D, Horwitz EM, Huben RP (2010). NCCN clinical practice guidelines in oncology: prostate cancer. J Natl Compr Canc Netw.

[28] Roach M, Hanks G, Thames H, Schellhammer P, Shipley WU, Sokol GH, Sandler H (2006). Defining biochemical failure following radiotherapy with or without hormonal therapy in men with clinically localized prostate cancer: recommendations of the RTOG-ASTRO Phoenix Consensus Conference. Int J Radiat Oncol Biol Phys.

[29] Akaike H (1992). Data analysis by statistical models. No To Hattatsu.

[30] Cooperberg MR, Freedland SJ, Pasta DJ, Elkin EP, Presti JC, Amling CL, Terris MK, Aronson WJ, Kane CJ, Carroll PR (2006). Multiinstitutional validation of the UCSF cancer of the prostate risk assessment for prediction of recurrence after radical prostatectomy. Cancer.

[31] Gronberg H, Damber JE, Jonsson H, Lenner P (1994). Patient age as a prognostic factor in prostate cancer. J Urol.

[32] Roxburgh CS, McMillan DC (2010). Role of systemic inflammatory response in predicting survival in patients with primary operable cancer. Future Oncol.

[33] Germano G, Allavena P, Mantovani A (2008). Cytokines as a key component of cancer-related inflammation. Cytokine.

[34] Hanahan D, Weinberg RA (2011). Hallmarks of cancer: the next generation. Cell.

[35] Schmidt H, Suciu S, Punt CJ, Gore M, Kruit W, Patel P, Lienard D, von der Maase H, Eggermont AM, Keilholz U (2007). Pretreatment levels of peripheral neutrophils and leukocytes as independent predictors of overall survival in patients with American Joint Committee on Cancer Stage IV Melanoma: results of the EORTC 18951 Biochemotherapy Trial. J Clin Oncol.

[36] Teramukai S, Kitano T, Kishida Y, Kawahara M, Kubota K, Komuta K, Minato K, Mio T, Fujita Y, Yonei T (2009). Pretreatment neutrophil count as an independent prognostic factor in advanced non-small-cell lung cancer: an analysis of Japan Multinational Trial Organisation LC00-03. Eur J Cancer.

[37] Sumbul AT, Sezer A, Abali H, Kose F, Gultepe I, Mertsoylu H, Muallaoglu S, Ozyilkan O (2014). Neutrophil-to-lymphocyte ratio predicts PSA response, but not outcomes in patients with castration-resistant prostate cancer treated with docetaxel. Int Urol Nephrol.

[38] Gueron G, De Siervi A, Vazquez E (2012). Advanced prostate cancer: reinforcing the strings between inflammation and the metastatic behavior. Prostate Cancer Prostatic Dis.

[39] Houghton AM, Rzymkiewicz DM, Ji H, Gregory AD, Egea EE, Metz HE, Stolz DB, Land SR, Marconcini LA, Kliment CR (2010). Neutrophil elastase-mediated degradation of IRS-1 accelerates lung tumor growth. Nat Med.

[40] Shamamian P, Schwartz JD, Pocock BJ, Monea S, Whiting D, Marcus SG, Mignatti P (2001). Activation of progelatinase A (MMP-2) by neutrophil elastase, cathepsin G, and proteinase-3: a role for inflammatory cells in tumor invasion and angiogenesis. J Cell Physiol.

[41] Petrie HT, Klassen LW, Kay HD (1985). Inhibition of human cytotoxic T lymphocyte activity in vitro by autologous peripheral blood granulocytes. Open J Immunol.

[42] Kao SC, Pavlakis N, Harvie R, Vardy JL, Boyer MJ, van Zandwijk N, Clarke SJ (2010). High blood neutrophil-to-lymphocyte ratio is an indicator of poor prognosis in malignant mesothelioma patients undergoing systemic therapy. Clin Cancer Res.

[43] Walsh SR, Cook EJ, Goulder F, Justin TA, Keeling NJ (2005). Neutrophil-lymphocyte ratio as a prognostic factor in colorectal cancer. J Surg Oncol.

[44] Shimada H, Takiguchi N, Kainuma O, Soda H, Ikeda A, Cho A, Miyazaki A, Gunji H, Yamamoto H, Nagata M (2010). High preoperative neutrophil-lymphocyte ratio predicts poor survival in patients with gastric cancer. Gastric cance.

